# Space Takes Time: Concentration Dependent Output Codes from Primary Olfactory Networks Rapidly Provide Additional Information at Defined Discrimination Thresholds

**DOI:** 10.3389/fncel.2015.00515

**Published:** 2016-01-14

**Authors:** Kevin C. Daly, Samual Bradley, Phillip D. Chapman, Erich M. Staudacher, Regina Tiede, Joachim Schachtner

**Affiliations:** ^1^Department of Biology, West Virginia UniversityMorgantown, WV, USA; ^2^Fachbereich Biologie, Philipps-UniversitätMarburg, Germany

**Keywords:** olfaction, spatial code, temporal code, spatiotemporal code, odor processing, principle neuron, psychophysics

## Abstract

As odor concentration increases, primary olfactory network representations expand in spatial distribution, temporal complexity and duration. However, the direct relationship between concentration dependent odor representations and the psychophysical thresholds of detection and discrimination is poorly understood. This relationship is absolutely critical as thresholds signify transition points whereby representations become meaningful to the organism. Here, we matched stimulus protocols for psychophysical assays and intracellular recordings of antennal lobe (AL) projection neurons (PNs) in the moth *Manduca sexta* to directly compare psychophysical thresholds and the output representations they elicit. We first behaviorally identified odor detection and discrimination thresholds across an odor dilution series for a panel of structurally similar odors. We then characterized spatiotemporal spiking patterns across a population of individually filled and identified AL PNs in response to those odors at concentrations below, at, and above identified thresholds. Using spatial and spatiotemporal based analyses we observed that each stimulus produced unique representations, even at sub-threshold concentrations. Mean response latency did not decrease and the percent glomerular activation did not increase with concentration until undiluted odor. Furthermore, correlations between spatial patterns for odor decreased, but only significantly with undiluted odor. Using time-integrated Euclidean distance (ED) measures, we determined that added spatiotemporal information was present at the discrimination but not detection threshold. This added information was evidenced by an increase in integrated distance between the sub-detection and discrimination threshold concentrations (of the same odor) that was not present in comparison of the sub-detection and detection threshold. After consideration of delays for information to reach the AL we find that it takes ~120–140 ms for the AL to output identity information. Overall, these results demonstrate that as odor concentration increases, added information about odor identity is embedded in the spatiotemporal representation at the discrimination threshold.

## Introduction

A fundamental cornerstone of sensory neuroscience is to understand internal representations of external stimuli. In nearly every sensory domain, this has been achieved by carefully relating neural responses of sensory neural systems to psychophysical measures of sensory function (Kandel, 2013). Surprisingly, although there has been considerable controversy about what “represents” an olfactory cue, almost no emphasis has been placed on establishing measures of olfactory representations at identified psychophysical limits of olfactory acuity. This absence of critically important relationship between psychophysical thresholds and physiological measures is unprecedented in sensory neuroscience.

Not surprisingly then, the relative contribution of spatial and temporal components of output responses from primary olfactory networks to the creation of salient olfactory percepts has long been highly controversial (Leon and Johnson, 2009). Olfactory sensory neurons (OSNs), which express the same receptor proteins (Buck and Axel, 1991; Clyne et al., 1999; Gao and Chess, 1999), project to the same glomerulus (or a pair of glomeruli in mammals) within the antennal lobe and olfactory bulb (AL/OB; Mombaerts et al., 1996; Wang et al., 1998; Vosshall et al., 2000). This input organization supports the proposition that odor identity is initially encoded as a spatial input pattern across glomeruli based on OSN tuning. Imaging studies in vertebrates and invertebrates show odor-dependent spatial patterns of glomerular activity (e.g., Joerges et al., 1997; Uchida et al., 2000; Meister and Bonhoeffer, 2001; Wachowiak et al., 2002; Hansson et al., 2003). These patterns are more or less stereotypical across individuals of the same species further suggesting that spatial input patterns are sufficient to explain olfactory “codes” (Galizia et al., 1999a; Rubin and Katz, 1999). However, with increased odor concentration, odor-dependent maps often expand and show increased excitation (Rubin and Katz, 1999; Fuss and Korsching, 2001; Sachse and Galizia, 2003; Strauch et al., 2012) and increasing concentration is often associated with better performance in behavioral discrimination tasks (Wright and Smith, 2004; Daly et al., 2008).

Furthermore, there is a nonlinear relationship between AL/OB input and output, indicating that local processing transforms the input signal (Wilson et al., 2004; Olsen et al., 2007); this takes time and relies on local network processing (Christensen et al., 1998a,b; Daly et al., 2011). As such, electrophysiological studies across a broad taxa demonstrate that individual principle output cells produce temporally complex responses that vary in the latency of excitatory response onset (Kauer, 1974; Kauer and Shepherd, 1977; Meredith, 1986). Furthermore, odor-driven neural ensemble responses are temporally dynamic (Friedrich and Laurent, 2001; Stopfer et al., 2003; Daly et al., 2004b; Krofczik et al., 2008), in *Manduca* evolving over ~120–140 ms (relative to onset of the excitatory response) in an odor dependent manner (e.g., Daly et al., 2004b; Staudacher et al., 2009) that correlates with psychophysical measures of odor discrimination (Daly et al., 2001a). Given that principle cells from within a glomerulus tend to spike synchronously (Christensen et al., 1998b, 2003; Schoppa and Westbrook, 2001), this suggests that the temporal pattern of responses at the level of glomerular output provides a neural basis for discrimination. Additionally, the time required to optimize odor dependent representations correlates with discrimination time in behavioral assays, independent of task difficulty (Ditzen et al., 2003; Uchida and Mainen, 2003; Budick and Dickinson, 2006; Krofczik et al., 2008; Wesson et al., 2008a).

Thus, AL representations are odor and concentration dependent and evolve rapidly. What remains unclear is how these representations change in relationship to shifts in perceptual salience. Specifically, what are the differences in spatiotemporal responses below vs. at or above an animal’s ability to detect and discriminate odors? This is an essential question precisely because spatiotemporal structuring of odor responses is concentration dependent. Thus, to understand the spatiotemporal requirements necessary to generate salient olfactory percepts, representations must be characterized within the context of stimulus intensities at and around the lower perceptual limits of the animal. To accomplish this, we used *Manduca sexta* moths in psychophysical and electrophysiological studies where we precisely matched stimulation protocols. We first identified detection thresholds for a panel of highly similar alcohols and ketones, then identified discrimination thresholds for a subset of odor pairs. We then compiled a virtual AL ensemble of intracellularly recorded projection neurons (PNs), which were registered to specific glomeruli. Each PN was presented the complete panel of odors below, at, and above identified detection and discrimination thresholds. By then characterizing odor representations as a function of both their spatial and spatiotemporal properties we determined that discrimination, but not detection threshold stimulus intensities significantly shifted spatiotemporal representations.

## Materials and Methods

### Experimental Animals

*Manduca* *sexta* moths were reared in a laboratory colony in the Department of Biology at West Virginia University as previously described (e.g., Tripathy et al., 2010; Daly et al., 2011). Larvae were reared on artificial diet (adapted from: Bell and Joachim, 1976). Throughout, larvae were kept in an incubator (Model 166VL; Percival Scientific) with a light:dark rhythm of 16:8 h; a temperature of 26.5°C; and a relative humidity of 40%. At pupal stage 17 individuals were placed in paper bags and transferred to a second incubator with a reversed 16:8 h light:dark cycle; 25°C and a relative humidity of 75%. In electrophysiological experiments, 6 to 8 day old adult males were used during the first 4 h of their dark cycle. Males were used because their ALs are more prominent and easier to impale. In all psychophysical experiments, both male and female adult moths between 5 and 8 days old were used (Daly et al., 2007, 2008).

### Odors

The current study used a panel of six monomolecular floral odor components, listed in Table 1 along with their abbreviated name, source, purity, density and vapor pressure. The selection of these odors was based on several criteria. First, *Manduca sexta* forage from a wide variety of plants (Fleming, 1970; Haber and Frankie, 1989; Mechaber and Hildebrand, 2000; Raguso et al., 2003b). As such they are able to detect and respond to a wide range of plant volatiles including odors such as 1-hexanol (A6), which is a compound of *Datura wrightii* (Raguso et al., 2003a) and a common floral component. Second classical conditioning experiments establish that *Manduca* can learn to respond selectively to all of these as well as many other odors (Daly and Smith, 2000; Daly et al., 2001a,b). Third, psychophysically defined detection and discrimination thresholds have been previously established for a subset of these odors (Daly et al., 2007, 2008, 2013) allowing us to make direct comparisons with the behavioral results herein. However, in the current study we precisely matched the stimulation protocols to those required for electrophysiology. Thus, by using this panel, we place the current study within the context of ecologically relevant odors that have been extensively characterized behaviorally.

**Table 1:** **Listing of all odors, their abbreviation, source, purity, density, molecular mass (MM) and vapor pressure (VP; mm Hg at 25°C)**.

Odorant	Abbreviation	Source	Purity (%)	Density	MM	VP
Alcohols
1-hexanol	A6	Sigma	97	0.861	102.17	0.928
1-octanol	A8	Sigma	99	0.820	130.23	0.079
1-decanol	A10	Sigma	97	0.890	158.28	0.009
Ketones
2-hexanone	K6	Sigma	98	0.810	100.16	11.600
2-octanone	K8	Sigma	98	0.820	128.21	1.350
2-decanone	K10	Sigma	98	0.824	156.26	0.248

### Olfactometer and Stimulus Parameters

**Figure 1:**
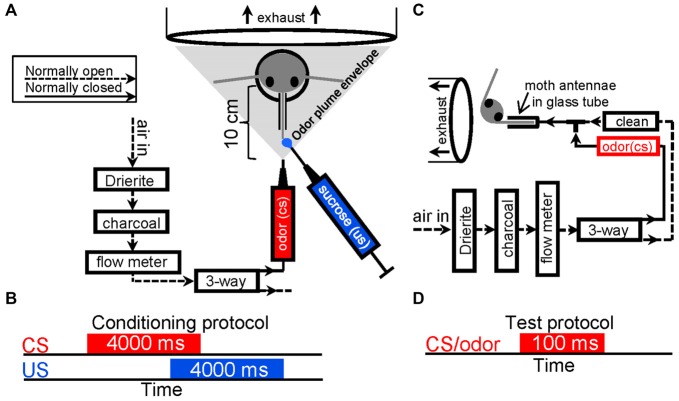
**Stimulus delivery protocols. (A)** Schematic of the odor delivery system with relationship to the moths head. Arrows indicate the path of airflow through the olfactometer. House compressed air was first passed through 500 cc’s Drierite and a 500 cc’s active charcoal before passing through a flow meter. From the flow meter, clean air then passed into a three-way valve then out of the normally open output unused. During conditioning, odor (the conditioned stimulus or CS) was presented to the antenna by actuating the valve and shunting airflow to the normally closed output, into an odor cartridge (red), then out of a 1 mm ID nozzle at a velocity of 3.3 m/s. The nozzle was positioned approximately 10 cm in front of the moth head; ejected odor spread creating a conical envelop (gray triangle) that covered and was pulled over the antennae and into an exhaust located behind the head at a measured velocity of 0.3 m/s. Sucrose solution (blue; the unconditioned stimulus or US) was presented manually to the partially extended and restrained proboscis using a Gilmont type syringe. **(B)** A schematic depiction of the CS-US timing during conditioning. **(C)** A modified version of the stimulus delivery system described in **(A)** used during behavioral testing and intracellular recordings. Here the normally open output from the three-way valve was tied into a T-fitting that also received the output of the normally closed output line, which contained the odor cartridge. The third branch of the T was then attached to a glass sleeve that was placed over a single antenna. Thus, clean or odor laden airflow was constantly passing over the antenna. All expelled output from the odor delivery system was captured in an exhaust vent. **(D)** Schematic depiction of the test stimulus duration.

Figures 1A,B show a schematics of the olfactometer design and how the moth was positioned during olfactory conditioning as well as stimulus timing. Briefly, after drying with Drierite (W.A. Hammond Drierite), compressed air was filtered with activated charcoal (C3014; Sigma), then, passed into three-way valve (LFAA1200118H; The Lee Company). Air passed out the normally open output line of the three-way valve and was not used. During odor stimulation the three-way valve was activated, shunting air into the normally closed output line and through an odor cartridge. The nozzle of the odor cartridge was placed 10 cm from the animal to insure bilateral dispersion over most if not all of the antennae. Figures 1C,D represent schematics of the olfactometer design used during both psychophysical and electrophysiological testing as well as the stimulation protocol used in each test trial. Here the same valve system was used but in this case the normally open output line was connected to the output nozzle as well as the normally closed output line. This insured that a continuous stream of conditioned air was always flowing over the antennae and that once dispensed it was immediately purged from around the antennae. In this case only a single antenna was exposed to odor; this antenna was placed into a glass tube to insure high temporal precision of odor delivery without mixing in the surrounding air. Prior to each experiment, airflow was measured with a flow-meter (ADM1000; Agilent) and adjusted to 250 ml/min, or a velocity of 3.3 m/s using an adjustable flow meter (PMR1–01293; Cole Parmer). This airflow velocity is well within the normal range of a flying moth in natural conditions and produces a 9 ms latency between valve actuation and odor reaching the antenna. Latency was calculated based on flow rate through the olfactometer and glass tube, and measured via fast photo ionization detection [200A miniPID; Aurora Scientific (Daly et al., 2013)].

We used custom-made odor cartridges with a volume of ~1.7 ml. Odor cartridges were either empty (i.e., blank), or they contained a small strip of filter paper (No. 1; Whatman International), which was loaded with 3 μl of either undiluted odor or diluted odor in mineral oil; thus each “concentration” is defined by the initial stimulus loading. For all experiments a 100 ms stimulus duration was used to mimic the time a moth is most likely to be exposed to odors when flying through naturally occurring odor plumes (Murlis and Jones, 1981). Other studies have used durations ranging from 20 ms in pulsed stimuli (Tripathy et al., 2010), 100 ms (Brown et al., 2005), 500 ms (Brown et al., 2005; Broome et al., 2006; Namiki et al., 2009), 1 s (Laurent et al., 1996; Sachse and Galizia, 2003; Stopfer et al., 2003; Mazor and Laurent, 2005) and 2 s (Joerges et al., 1997; Faber et al., 1999; Galizia et al., 2000; Friedrich and Laurent, 2001; Galán et al., 2004) to 10 s in locust (Mazor and Laurent, 2005). Shorter odor stimuli in the range of <300 ms have been shown to elicit short bursts of spikes from responding PNs, while spiking patterns increase in complexity and in particular variability when stimulus duration is on the order of 1 s or longer (Christensen et al., 1998b). For electrophysiological studies, each stimulus was repeated five times, each separated by 10 s of clean air thus a single cartridge was used for 500 ms. Blank stimuli were presented first followed by each sequential increase in concentration in the dilution series and this was always from low to undiluted (neat). Within each concentration (or loading), all odors were presented in a pseudorandom sequence. The approach of presenting all odors at each concentration from low to high reduces possible adaptation effects, which are particularly expected at the very highest concentrations. For the psychophysical studies we used a five log step dilution series ranging from 0.001–100.0 μg/2 μl for the detection threshold experiments, a three log step dilution series for discrimination thresholds from 0.1–100 μg/2 μl and based on the behavioral results, we used three dilutions from this series (0.001, 0.01, 1.0 μg/2 μl) as well as undiluted odor (~1640 μg) for electrophysiology and in all cases odor cartridges were used for a single 100 ms trial. Finally, in all experiments, odor cartridges were loaded and sealed at least 1 h prior to use.

Finally, it is worth noting that there are several factors, in addition to the molecular features of an odorant, which affect the actual concentration of odorant dispensed and subsequently interacting with the antennae. These factors include, the solvent/diluent used, temperature, the substrate that the odorant is loaded onto, airflow and the specifics of the olfactometer used to name a few. Our overall approach was designed to produce rapid brief and highly repeatable stimulus delivery directly to the antenna, we note that these design requirements affect the final stimulus delivery. Nevertheless, our prior work has established that this commonly used approach does deliver a systematic increase in concentration as measured at the level of mass antennal input and behavioral response (Daly et al., 2007).

### Psychophysical Determination of Detection and Discrimination Thresholds

In order to behaviorally assess olfactory acuity, moths were first conditioned using well established protocols (Daly and Smith, 2000). Figure 1A schematically depicts the methodological approach to olfactory conditioning. Briefly, moths were restrained in tubes and impaled with an EMG electrode in the cibarial pump muscle (the primary feeding muscle) and a reference in the contralateral eye. The proboscis was restrained so that timing of the unconditioned stimulus (5 μl of 0.75 m sucrose manually applied to the restrained proboscis tip) was possible. For detection threshold studies, all six odors from the panel (Table 1) were used (*N* = 60 moths per odor). Moths were first conditioned with a single target odor (conditioning stimulus or CS) followed by food so that they learned to elicit a feeding response to the target odor. Odor stimulation during each conditioning trial lasted 4 s and was overlapped by 1 s with 4 s of 0.75 M sucrose solution (Figure 1B). This allows a 3 s window for the moths to elicit a conditioned response (CR) for each trial. Subsequently at both 24 and 48 h post conditioning, moths were tested with a dilution series of the target odor from low to high concentration using the stimulation protocols depicted in Figures 1C,D. If moths elicited a conditioned feeding response from the cibarial pump muscle within 7 s of odor onset, it was recorded as a conditioned feeding response; from these data the probability of a CR was calculated by taking the percentage of moths producing a CR and dividing by 100 (Daly et al., 2007).

We also performed discrimination threshold studies for a subset of odor pairs. We selected four of the fifteen possible odor pairs (A10 vs. K6; K10 vs. A6; A10 vs. K10; and K6 vs. K8; see Table 1). These combinations allowed us to measure discrimination thresholds for the most and least similar odor combinations; in this case similarity was based on the molecular features moiety and carbon chain length as well as the most and least volatile odors. During conditioning both odors were presented in the same manner but one was reinforced with sucrose solution (CS+) as shown in Figure 1A and the second was not (CS−). CS+ and CS− trials were pseudo randomized using two sequences to avoid sequence effects. A total of 60 moths were used in each of four differential conditioning experimental groups. Within each group, the CS+ and CS− (i.e., reinforcement contingencies) were experimentally counterbalanced such that half of the animals received one odor as the CS+ and the other half received the second odor as the CS+; these data were then averaged (Daly et al., 2001b, 2008). At 24 and 48 h moths were pseudo randomly tested across a log step dilution series of both the CS+ and the CS−. Odors were always presented from low to high concentration and activation of the cibarial pump muscle served as an indicator of a CR. As with the detection threshold data, CR percentages were converted into CR probabilities. For display purposes, a discrimination index was calculated by quantifying the difference between CR probability CS+ and CS− for each stimulus dilution. Statistical comparisons between the blank responses and responses to each concentration served as a means to statistically determine detection thresholds whereas statistical comparison between CS+ and CS− within each concentration served as a means to determine discrimination threshold for odor pairs. All tests were one-tailed paired *t*-tests using *p* < 0.05 as the statistical threshold; data from 24 and 48 h tested were statistically assessed independently and based on common threshold values across odors results were averaged for display purposes.

### Intracellular Recording and Staining

Dissections were previously described (Christensen and Hildebrand, 1987; Staudacher et al., 2009). Specimens were constantly superfused with *Manduca* saline (Heinbockel et al., 1998). Brains were exposed by removing all cuticle between the compound eyes and antennae, removing tracheae, connective tissue and musculature. For easier penetration of the glass microelectrodes, a small patch of neurilemma was removed from the AL. Borosilicate glass (OD 1.0 mm, ID 0.5 mm; BF100-50-100; Sutter Instruments) microelectrodes were produced using a horizontal puller (P-2000; Sutter Instruments). Electrode tips were filled with 5% Neurobiotin and the stems with 2M potassium actetate and connected to the amplifier with a silver/silver chloride wire. Electrode resistances were between 190–280 MΩ. A second chloride silver wire under the brain was used as reference electrode. Neural signals were amplified with an Axoclamp 2B amplifier in bridge mode (Molecular Devices). After A/D conversion (Digidata 1440A; Molecular Devices), signals were written to a PC hard disk (16 bit at 10 kHz; Clampex, version 10.1; Molecular Devices).

### Histology and Identification of Glomeruli

Upon completion of the experimental protocols, the brains were excised and fixed with 4% formaldehyde in Millonig’s buffer then removed from the head and stored in Millonig’s buffer. Brains were embedded in agarose (BP160–500; Fisher Scientific) and 70 or 240 μm vibratome sections were made (VT1000S; Leica). Neurobiotin was detected with either Avidin-Texas Red (A820; Molecular Probes) or Streptavidin-CY3 (016-160-084; Jackson ImmunoResearch). Stained sections were coverslipped with Permount (SP15-500; Fisher Scientific). Brain sections were scanned with a confocal-laser-scanning microscope (BX61 with a FV1000 scan unit and FV10-ASW software; Olympus) using either a 10×/0.40 or a 20×/0.75 objective (UplanSApo; Olympus). Glomerular structure was visualized with background autofluorescence. Stacks of tagged image files were exported for later use with AMIRA (version 4.1; Visage Imaging).

To determine, the glomerulus each PN arborized in, a three-dimensional AL atlas was used (El Jundi et al., 2009). Image stacks were imported into AMIRA and aligned to the reference atlas (Huetteroth and Schachtner, 2005). The glomerulus that a given PN arborized within was determined by comparing the confocal data with the reference atlas. For reference, the soma cluster that each PN cell body was located in and the output tract the axon projected through was determined. Because our stimulation protocol always proceeded from low to high concentration, all recordings with partial records were typically missing higher concentration stimuli making comparisons with them impossible. Therefore, we only used those PNs from our database for which responses to all odors and concentrations were attained (~43 min recording time) and for which staining allowed identification of the glomerulus that the PNs dendritic tree arborized. Thus, of 125 recorded PNs, 17 uniglomerular PN1a type neurons (Matsumoto and Hildebrand, 1981) each arborizing a unique glomerulus, met this criterion and represent the population analyzed herein. By selecting only completed recordings, direct comparisons of odors and concentrations can be performed without making assumptions about the consistency of responses when using multiple incomplete records from the cells of the same glomerulus.

### Assembling the Virtual Ensemble

First, all recorded spike trains in response to all stimuli from the 17 PNs were aligned with respect to stimulus-onset. This allows odor representations of this virtual PN ensemble to be analyzed as if recorded simultaneously in a single prototypical AL (Daly et al., 2004a; Staudacher et al., 2009; Houot et al., 2014). This method has been pioneered and successfully applied in other systems (e.g., Georgopoulos et al., 1988; Ruiz et al., 1995; Georgopoulos, 1996; Skaggs and NcNaughton, 1998) and termed a “virtual ensemble” (Skaggs and NcNaughton, 1998).

We treated odor responses of single PNs as representative of their associated glomerulus as previously established (Namiki and Kanzaki, 2008; Staudacher et al., 2009). There are 63 glomeruli and ~900 PNs (Anton and Homberg, 1999; Schachtner et al., 2005) meaning on average ~12–14 PNs arborize within each glomerulus. It has been shown that neurons arborizing within the same glomerulus have very similar response patterns (Reisenman et al., 2005; Namiki et al., 2009) and furthermore tend to fire synchronously as shown in paired intracellular recordings in both insects and mammals, though this does not imply that all PNs within a given glomerulus are redundant (Schoppa and Westbrook, 2001, 2002; Lei et al., 2002). Nevertheless, the overall virtual ensemble produced herein represents ~27% coverage of all male *Manduca* AL glomeruli. By comparison, in imaging studies on *Apis* and *Drosophila* ~11–22% of their respective AL glomeruli are typically recorded (Faber et al., 1999; Galizia et al., 1999c; Sachse et al., 1999; Sachse and Galizia, 2003; Silbering and Galizia, 2007; Silbering et al., 2008). Thus although our virtual ensemble is reasonably small, our approach is unique in that for each cell we have a comprehensive record of responses to all odors and concentrations while providing as good or better spatial coverage than a typical imaging study in insects but with spike time resolution.

### Analysis of Spike Count, Maximum Spike Frequency and Response Latencies

One preliminary question was whether these odors and concentrations produced differences in the massed response of the virtual ensemble, where both spatial and temporal information are essentially removed, or whether the differences were encoded within the detailed responses of individual cells. Thus, the goal of this first analysis was to confirm that the magnitude of the summed output could not explain odor-dependent and/or concentration-dependent differences in stimulus-driven population responses but rather that this information was encoded in the details of individual responses across the population. All analyses were performed with custom written functions in Matlab (version 2007b, Mathworks, Inc.). Time stamped data were used to generate peristimulus rasters, generate stimulus-driven spike counts, and calculate maximum instantaneous firing frequency. These calculations were performed for each stimulus-driven response. Spike count and maximum instantaneous spike frequency (the inverse of the time (s) between two spikes within the sampling window) measures were used as a basis for statistical analysis of differences in responses as a function of odor, concentration and their interaction. Calculations were based on a peri-stimulus time window between −500 and 1500 ms.

A Jarque-Bera test indicated non-normally distributed spike train data (*p* < 0.001). Therefore, we used the a generalized mixed linear model (GLIMMIX) procedure, which allows for non-normally distributed categorical and continuous variables (SAS, version 9.1.3; SAS Institute, Inc.; Schabenberger, 2005). Concentration was treated as a continuous predictor. PN identity and odor identity were treated as categorical predictors. Furthermore, PN identity was modeled as a random predictor. Third order polynomials were fit to mean spike counts and instantaneous frequency measures as a function of concentration. Unless stated otherwise, data were plotted as mean ± standard error of the mean. Finally all *post hoc* comparisons were Kruskal-Wallis tests, using *p* < 0.01 as the significance threshold and Bonferroni corrections for multiple comparisons.

To determine the degree to which the recorded population could produce odor and concentration-dependent spatial and temporal response patterns, the response of individual PNs to each stimulus was categorized as excitatory, inhibitory or non-responsive. Here, Spike counts were *z*-score normalized and classified and responses were classified as inhibited (*z-score* ≤ −0.5 *SD*), non-responsive (−0.5 < *z-score* < 2.0 *SD*) and excited (*z-score* ≥ 2.0 *SD*). In this case *z*-scores ≤ −0.5 *SD* are interpreted as inhibited because these values typically corresponded to inhibitory subthreshold events in the raw voltage traces as well as a consistent spike suppression prior to spike bursting (Staudacher et al., 2009). Results were averaged across odors and repeats and summarized by response class as a function of concentration.

Finally, to calculate response latencies, we first identified the first pair of spikes elicited after stimulus onset that produced an ISI < 30 ms and measured the time from stimulus onset to the first spike of the pair. The <30 ms threshold was chosen after manual inspection of results, which indicated that this ISI correctly ignores spontaneous spiking that typically occurs prior to onset of the initial I_1_ inhibitory phase of the response and accurately identified the initiation of the subsequent excitatory response. Latencies where then statistically compared using a series of one-tailed paired *t*-tests with a significance threshold of *p* < 0.05.

### Correlation of Spatial Patterns

The above *z*-score transformed spike counts were also used as a basis for correlating spatial response patterns between different odors as a function of concentration. This analysis allowed us to determine whether spatial representations become less correlated as a function of increasing concentration. We calculated the Spearman’s rank correlations for all possible pair wise combinations of odors at each concentration. We again used a −500–1500 ms peristimulus window. To maintain a purely spatial representation, we classified responses into three the response types; excited, inhibited and non- responsive based on the methods described above. Finally, we used Kruskal-Wallis tests (ρ = 0.01 with Bonferroni correction) as a basis to determine whether the between odor spatial correlations were significant at different concentrations; in this case however, a non-significant correlation indicates a statistically distinct spatial representation (i.e., a decorrelation).

#### Euclidean Distance (ED) Analysis

Finally, to quantify spatiotemporal differences in odor representations, ED analysis was used (Stopfer et al., 2003; Daly et al., 2004b; Brown et al., 2005; Staudacher et al., 2009). This method quantifies differences in spatial patterns of odor driven spiking responses across the recorded ensemble (i.e., PN/glomerulus identity) as a function of time (*z*-score normalized spike counts per time bin). Data were analyzed across a peristimulus time window from −0.5–1.5 s using 20 ms bins. The ED analysis treats each time bin as a single point in a multi-dimensional space. The dimensionality of this space is defined by the number of neurons in the virtual ensemble. The Pythagorean Theorem was used to calculate the straight line (i.e., shortest) distance between ensemble representations of two stimuli at each bin in time; because each neuron is independently recorded, they represent orthogonal dimensions. Thus each step in time provides an accurate measure of the distance between representations of any given pair of stimuli that the ensemble responded to. All possible pairwise comparisons were calculated for within-stimulus (i.e., repeats of the same stimulus) and between stimuli (i.e., comparisons between different odors or concentrations including blank responses). Resultant ED measures were averaged and then normalized by subtracting the corresponding mean within-stimulus-repeat ED values. Thus, spontaneous activity varied around 0 *SD*s and values ≥2 *SD* can be considered significant differences between comparison representations. Finally, we calculated the integrated distance from these normalized ED values starting at stimulus onset and summing successive distances values.

## Results

### Similar Odors have Similar Thresholds

**Figure 2:**
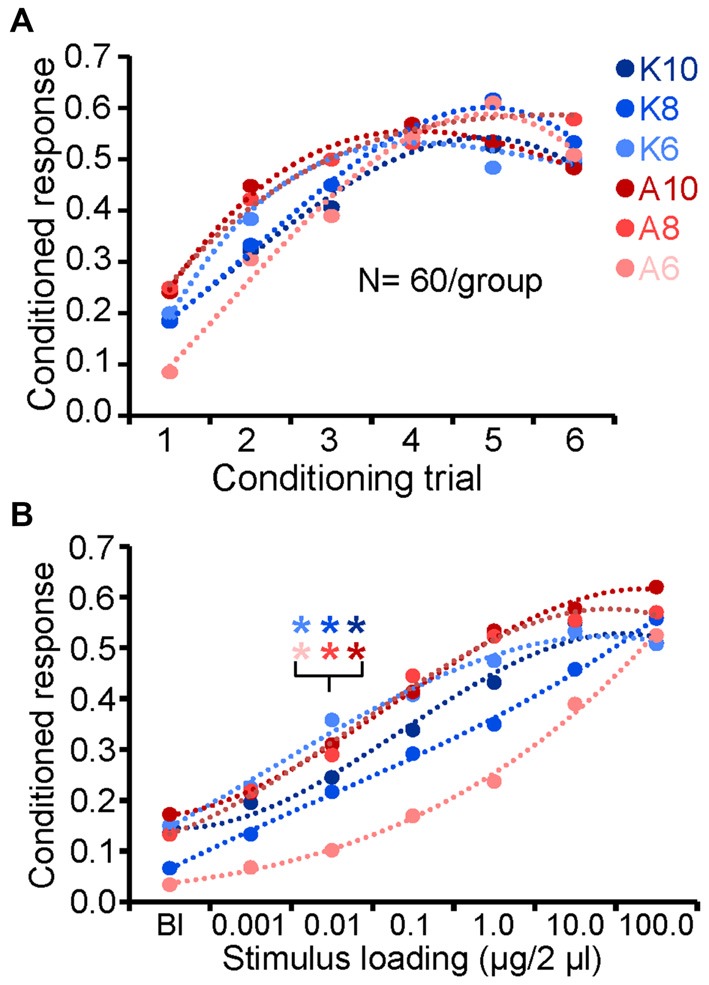
**Behavioral identification of detection thresholds. (A)** Acquisition of the conditioned feeding response probability for each monomolecular odorant. Odors are color coded to identify moiety (ketones = blue; alcohols = reds) and shaded to indicate carbon chain length (dark = 10; medium = 8; lite = 6). **(B)** Conditioned feeding response probability as a function of odor stimulus loading (concentration). Results based on the average across testing days. Statistical comparison of the conditioned response (CRs) for each CS concentration with the corresponding blank (zero odor) was used to identify detection thresholds (indicated by inset asterisks; one tailed paired *t*-test; *p* < 0.05).

Our overall goal was to characterize spatiotemporal neural representations of odors at and around detection and discrimination threshold limits. Thus we characterized detection thresholds for all odors and discrimination thresholds for a subset of odors. For all odors, acquisition of the CR peaked by the fourth or fifth conditioning trial which is consistent with previous results (Figure 2A; Daly et al., 2001a, 2007, 2013). Figure 2B highlights the mean response to the blank and each concentration of odor during the test phase of the experiment. Within group statistical comparisons of responses to the blank and responses to subsequent concentrations of CS indicated that 0.01 μg/2 μl of odor produced a significant increase in CR probability for all odors (one tailed paired *t*-test; *p* < 0.05). This finding was consistent across all odors when tested at both 24 and 48 h post conditioning except for K10, which produced significant thresholds at 0.01 and 0.1 μg/2 μl at 24 and 48 h posttests respectively. We then pooled both posttest days for K10 and reanalyzed. In this case, 0.01 μg/2 μl was significant and hence regarded as the detection threshold. Overall, these consistent results should be somewhat expected as these are highly similar odors in many (carbon chain length, moiety, masses and densities) but not all (vapor pressure) physical features (see Table 1).

**Figure 3:**
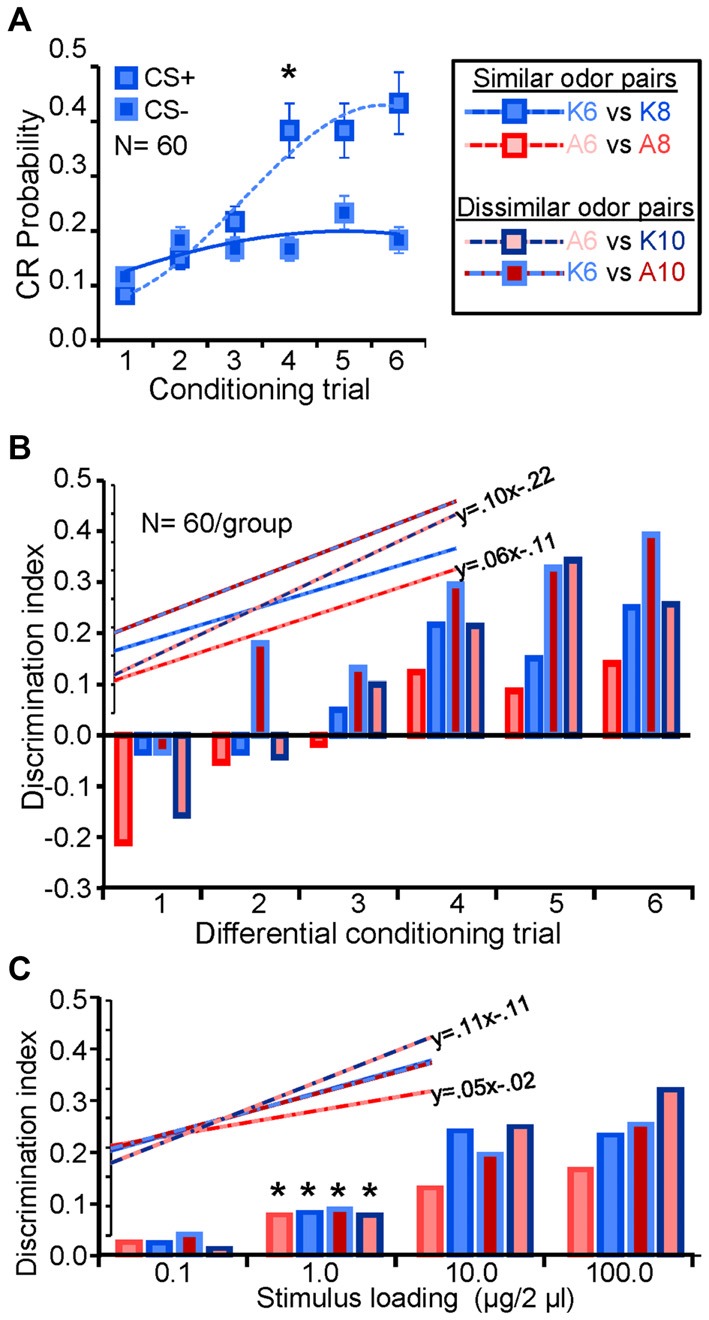
**Consistent detection and discrimination thresholds. (A)** Acquisition of the differential CR for K6 and K8. Both odors were used as the CS+ and CS− odorants and results were pooled as part of a counterbalanced design, hence results are presented as CS+ and CS−. Inset asterisks indicate the trial at which the CS+ and CS− produced a significant differential response (one tailed paired *t*-test; *p* < 0.05). Error bars represent the standard error. **(B)** Discrimination index as a function of successive conditioning trials for all four odor pairs used in this study. Bar outline and fill are color coded to identify the moiety (ketones = blue; alcohols = reds) and shaded to indicate carbon chain length (dark = 10; medium = 8; lite = 6) of the odorant pair. The discrimination index displays the mathematical difference between the CS+ and CS− responses by trial. Note that the initial negative values occur during conditioning because half of all of the first CS− conditioning trails were preceded by a CS+ trial which generalizes to the first CS− trial (see Daly et al., 2001a). Inset are the linear regression functions for each odor pair with the linear regression equations for the steepest and shallowest regression function; this highlights that A6 vs. A8 was the most difficult differential conditioning task to learn whereas A6 vs. K10 was the easiest. **(C)** Discrimination index as a function of stimulus loading (concentration) during the testing phase. The discrimination index in this case is the mathematical difference between the CS+ and CS− responses at each test loading. Results averaged across testing days. Significant difference between CS+ and CS− are highlighted by asterisks and indicate the lowest loading at which a significant differential response between the CS+ and CS− occurred (one tailed paired *t*-test; *p* < 0.05). Inset are linear regression functions for each odor pair as a function of loading. Also inset are corresponding regression equations for the steepest and shallowest functions, highlighting that A6 vs. A8 was the most difficult odor pair to discriminate whereas A6 vs. K10 was the easiest.

Next we generated discrimination thresholds for a subset of odor pairs: K6 vs. K8; A6 vs. A8; A6 vs. K10; and K6 vs. A10. These odors represent most and least similar odor pairs, as well as most and least volatile odors in the set. Figure 3A displays the acquisition of the conditioned differential response for one odor pair (K6 vs. K8), which shows a typical progression from highly similar responses in the first 2–3 differential conditioning trials (Daly et al., 2008). This progression can be quantified by generating a discrimination index that represents the difference between the CS+ and CS− responses (i.e., CS+–CS−). Using this index, Figure 3B demonstrates that across the four odor pairs, differential conditioning resulted in a systematic increase in the differential CR. Linear regression of this index as a function of conditioning trials furthermore indicates that acquisition of the differential response occurred at approximately the same rate albeit with differing intercepts, suggesting that A6 vs. A8 was a relatively more difficult discrimination learning task (Figure 3B, inset). Discrimination threshold testing at 24 and 48 h indicated consistent results across the odor pairs with 1.0 μg/2 μl producing a significant differential response on both test days for all but one odor pair (A6 vs. A8); in this case 1.0 μg/2 μl produced a significant differential response at 48 h but not at 24 h (which was significant at 10 μg/2 μl). However, when pooled across test days the discrimination thresholds for all four odor pairs occurred at 1.0 μg/2 μl (*p* < 0.05; Figure 3C). Figure 3C also suggests that A6 vs. A8 was a more challenging task for the moths as indicated by a relatively shallow slope across concentration, whereas A6 vs. K10 was the easiest as indicated by a relatively steeper slope (Figure 3C, inset); overall, these differences appear nominal.

### Peristimulus Rasters Indicate Odor and Concentration Dependent Spatial and Temporal Components

**Figure 4:**
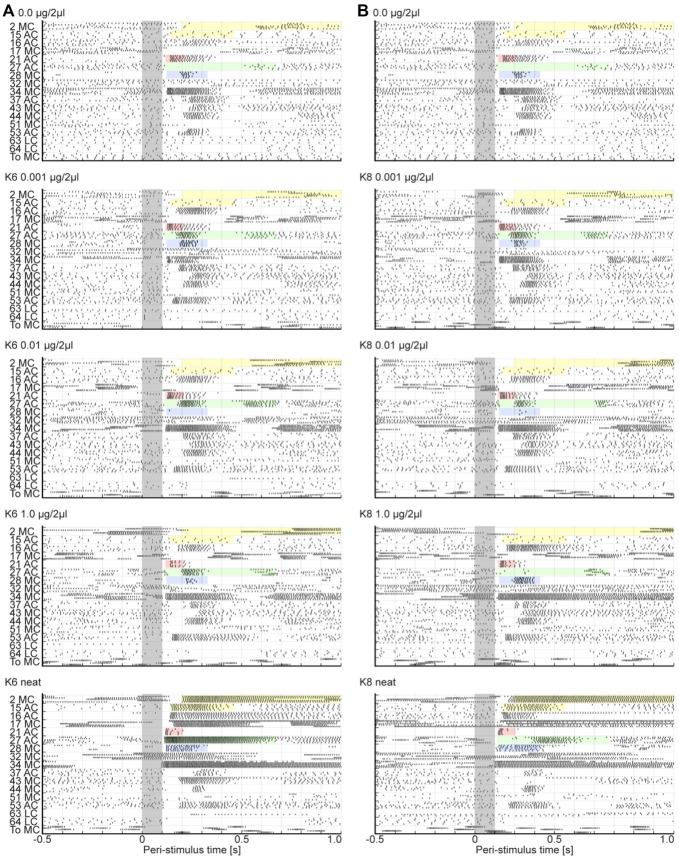
**Raster plots show the different PN responses types.** Peri stimulus raster plots show the responses for 17 uniglomerular Projection neurons (PNs) to four stimulus loadings of K6 **(A)** and to K8 **(B)**. The blank raster (top panel) is provided above each odor column in order to facilitate comparison of subtle differences in temporal details of each response. Each PN is listed on the ordinate and was named based on its glomerular association and the soma cluster from which the PN originated. For example the cell “2 MC” has dendrites in glomerulus 2 and its cell body in the medial cluster. All PNs exited the AL via the inner antenno-cerebral tract and arborized in MB calyx before terminating in the lateral protocerebrum. For each PN, five rows of hash marks represent the spikes of the five stimulus repeats. Note that all PNs had axons in the IACT. The gray bar marks the stimulus, which represents time zero. Inset coloration highlights exemplar responses. Each highlighted area in all panels corresponds to the start time and approximate duration of the response to neat K6 (bottom left most panel). This facilitates direct comparisons of responses from specific cells across loadings and odors. Abbreviations: AC, LC, MC: anterior, lateral and medial soma cluster, respectively; To: toroid.

Based on the psychophysical results above, we selected odor concentrations for use in the electrophysiology experiments that would represent sub-detection threshold (0.001 μg/2 μl), detection threshold (0.01 μg/2 μl), and discrimination threshold (1.0 μg/2 μl) and above discrimination threshold (undiluted or “neat”). The compiled virtual ensemble of 17 uniglomerular PNs, all had a comprehensive recording of five responses each to a blank, the three concentrations of the dilution series, and undiluted odor across all six odors used (150 stimuli). We also identified the glomerulus that each PN’s dendrites arborized. These data therefore contain information about the spatial pattern of AL responses and information about response onset, duration and strength with millisecond temporal resolution. Since the dilution series contains concentrations below, at and above identified thresholds, comparison of these concentrations with responses to blanks provides information about the spatial and temporal components that may be present at detection thresholds but not below. Furthermore, this virtual ensemble also provides information about the spatial and temporal components that may be present at discrimination thresholds but not below as well as what can be produced in excess with very high concentrations. Figure 4 displays peristimulus rasters of the virtual ensemble in response to the blank (top panels) and the dilution series for K6 and K8 (left and right columns respectively). Note that blank responses are attributable to mechanical artifacts generated by the olfactometer and not contamination as determined by both hotwire anemometry, which shows that valve actuation creates a pressure wave, and photoionization studies, which show no measureable levels of odorant present in the blank stimulus (Daly et al., 2013). In mammals, this mechanosensory component of the response is mediated by the G-protein coupled receptors themselves (Connelly et al., 2015).

PNs of the virtual ensemble produce spatially and temporally heterogeneous responses that are both odor and concentration dependent, and range from prolonged inhibition to excitatory bursting responses with differing onset latencies. Bursting responses however have consistent onset latencies within a given set of stimulus repeats. To highlight this heterogeneity, inset color-shaded areas are time aligned to the start and duration of the respective PN in response to neat K6. Shaded in yellow, for example, are two cells that were broadly inhibited to the blank and the dilution series of both K6 and K8. However, in response to undiluted odor, both cells elicited a strong and prolonged tonic spiking response with different onset latencies that were odor dependent. Here cell 2 MC consistently initiated an excitatory response at 200 ms post stimulus onset for neat K6 and about 160 ms post stimulus onset for neat K8. Thus this PN exhibited not only a concentration dependent effect but also exhibited a difference in onset latency for different odors. Not all cells increase response with concentration however. Highlighted in pink, is a cell (21 AC) that responded to the blank and both odors at all concentrations but the magnitude of the response appeared to decrease with concentration. Furthermore while the onset latency (the latency of the first spike for a given stimulus) within repeats of a given stimulus varies by no more than ±2 ms, the onset latency between stimuli is heterogeneous, varying by as much as about ±15 ms. While this difference in onset latency is subtle in this PN, stimulus-specific differences in onset latency can be far more profound. For example, highlighted in green and blue are two PNs (27 AC and 28 MC respectively). These two PNs produced excitatory responses with odor and concentration dependent onset latencies that varied from ±55 ms up to ±110 ms respectively. Furthermore they exhibited responses ranging from inhibition (i.e., dropping out of the response ensemble), to brief 50–100 ms excitatory bursts, to tonic excitatory responses. Finally, in response to neat odorant they exhibited prolonged duration responses. This overall description of responses is consistent with previous reports of response types in this model system (Christensen and Hildebrand, 1987; Christensen et al., 1998b; Staudacher et al., 2009), suggesting that this virtual ensemble is representative of typical single cell and multi-unit data. Thus, within our virtual ensemble are individual PNs, which have been addressed to specific glomeruli and which exhibit a variety of stimulus specific spatial and temporal response patterns.

### Odor and Concentration Dependent Effects are not Evident when Space and Time are Collapsed

**Figure 5:**
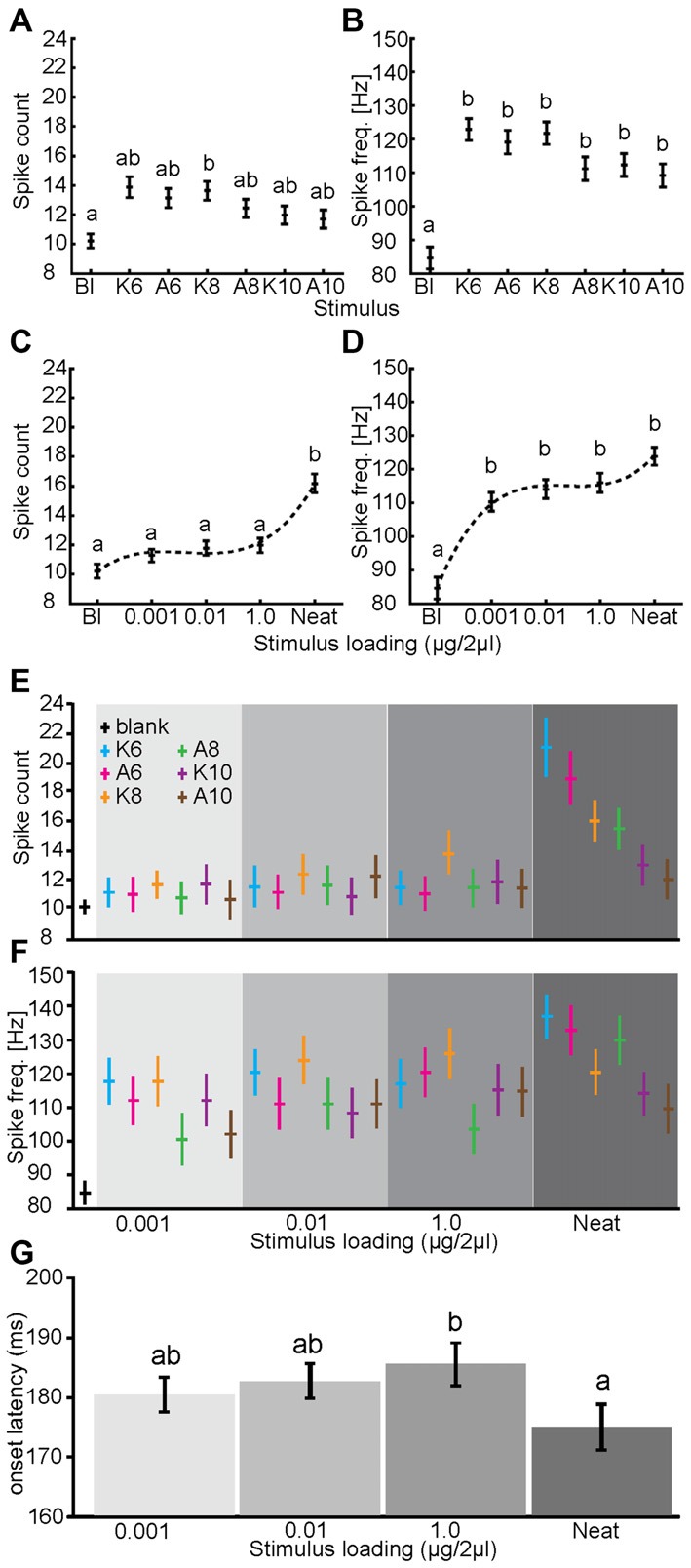
**Massed activity provides odor dependent and limited concentration dependent information.** The main effects of odor identity **(A,B)** and stimulus loading **(C,D)** on spike counts **(A,C)** and peak spike frequency **(B,D)**. For this analysis spatial and temporal information are collapsed. By comparison to blank, all odors produced a significantly higher peak spike frequency (**B**; *p* < 0.01; Kruskal-Wallis) but not significantly different spike counts **(A)** though the trend was consistent with frequency. Note that there are no significant between-odor differences in either count or frequency. In response to different concentrations of odor stimulation, neat odor presentation resulted in a significantly greater overall spike count (**C**; *p* < 0.01) but not peak spike frequency. Conversely, blank stimuli resulted in significantly lower peak spike frequency than did odor at any stimulus loading (**D**; *p* < 0.01). **(E,F)** The two-way interactions of odor and stimulus loading on spike counts and peak spike frequency. Note in both measures that neat odor did produce some odor dependent effects, these appear to relate to odor volatility. **(G)** Mean response onset latency relative to stimulus onset (in ms) as a function of stimulus loading (blank responses dropped). Inset letters indicate statistical differences in latency (one-tailed paired *t*-test; *p* < 0.05). In all panels, error bars represent standard error.

Visual inspection of the virtual ensemble responses in Figure 4 suggests that there is considerable heterogeneity in both spatial and temporal components of odor driven responses that could provide information to the animal about the presence and identity of an odor. This does not exclude the possibility that differences in the magnitude of the virtual ensembles response might account for odor and concentration dependent differences associated with either odor detection or discrimination. This effect should be considered a potential confound for subsequent spatial and spatiotemporal analyses. Thus, we first sought to confirm that the output of this specific sub population, when both the spatial and temporal response heterogeneity is collapsed, cannot account for effects around the detection and discrimination thresholds. To address this possible confounding effect, response magnitude was measured as both number of spikes (in a −500–1500 ms response window) and peak instantaneous spike frequency (i.e., the minimum interspike interval) for each PN response and statistically analyzed. Results of this analysis are displayed in Figure 5.

First, the main effect of odor significantly impacted spike counts (*p* < 0.01; GLIMMIX). However, although the response to the blank (treated as a separate stimulus in this analysis) appears substantially different from odor driven responses, as shown in Figure 5A, inset *post hoc* analysis indicates that the only difference was between the blank and K8 (*p* < 0.05; Kruskal-Wallis). The main effect of odor also significantly impacted peak frequency (Figure 5B). Here *post hoc* analysis revealed that while all odors elicited approximately the same peak spike frequency, they were all significantly above that elicited by the blank. This indicates that the magnitude of the olfactory response, while distinct from a purely blank response (averaged across concentration), is not statistically different as a function of the odor presented. Similarly, the main effect of concentration also significantly affected spike counts and peak spike frequency (*p* < 0.01; GLIMMIX). Inset *post hoc* analysis of concentration indicated that only neat odor elicited a significant increase in spike counts relative to the blank (Figure 5C) while all concentrations of odor elicited a significantly higher peak spike frequency than the blank, different concentrations of odor did not yield significant differences (Figure 5D). Consistent with prior results the AL also responded to odors at concentrations lower than what is observed in psychophysical assays of detection (Daly et al., 2007). Finally, the two way interaction of odor by concentration also significantly affected both measures of response magnitude (*p* < 0.01; GLIMMIX). As shown in Figures 5E,F this interaction indicates that primarily undiluted six and eight carbon alcohols and ketones account for the significant effect of concentration on spike counts. To provide an indication that there is information available within the spatial distribution of the response, we assessed the two and three-way interactions of PN identity with both odor and concentration. All three of these interactions were significant (*p* < 0.01; GLIMMIX). Results of the two-way interactions imply that the response magnitude of individual PNs was dependent on both the odor and the concentration it was presented. Consistent with the raw peri stimulus rasters shown in Figure 4, the three-way interaction indicates that the response of PNs to odor, and the changes in those responses as concentration changed, was idiosyncratic. Finally, comparison of response latencies demonstrates that across the dilution series there are no significant changes in latency except undiluted odor, which shows a significant ~10 ms decrease in response latency relative to the highest dilution. Although not significant, more diluted odors had lower latencies then the discrimination threshold, suggesting that if information is present at the discrimination threshold, it likely takes a few milliseconds more to emerge.

Overall, these analysis indicates that when the virtual ensemble data is collapsed by space and time, there is little if any measurable information remaining that could be used by the moth to determine odor identity particularly at psychophysical threshold concentrations as all of the statistical effects were either by comparison with the blank or neat odorant. Furthermore, there is limited information in the massed responses from which the moth can determine stimulus concentration. In this case the two way interaction of odor and concentration indicates that only highly volatile odors at undiluted concentrations provide significant information that the moth might use. We note however that undiluted odor represents approximately a five log step increase in concentration relative to the discrimination threshold (1.0 μg/2 μl). Importantly, these results cannot account for the identified detection or discrimination thresholds shown in Figure 3. Finally, it is clear that undiluted odor produces responses that are distinct from peri-threshold concentrations based on stronger, spatially greater and more decorrelated spatial patterns that begin to emerge earlier in response time.

### Each Stimulus Elicits a Distinct Glomerular Response Pattern

**Figure 6:**
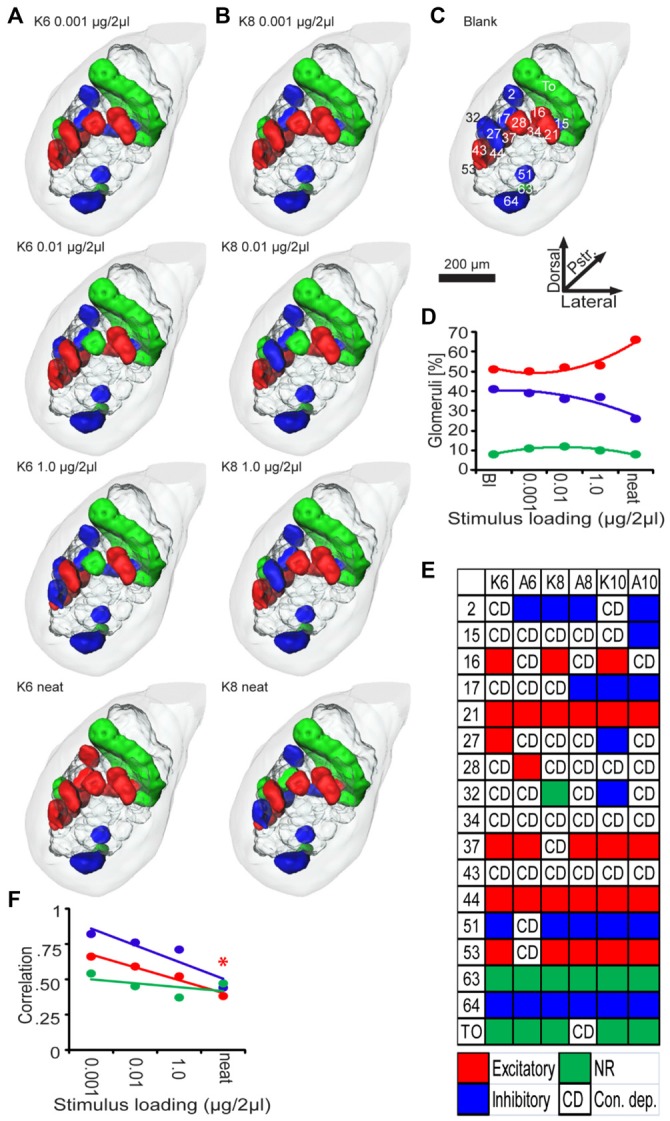
**Odor and concentration dependent patterns of glomerular activity.** Three-dimensional representations of AL responses to K6 **(A)** K8 **(B)** presented as a function of stimulus loading (in columns). **(C)** Three-dimensional representations of AL responses to the blank (no odor) stimuli. Glomeruli are color coded according to their *z*-score normalized spiking response during 0–500 ms peri-stimulus time window. Inset numbers and letters in **(C)** are the glomerular designations. Note that in response to different loadings of K6 or K8, a unique combinatorial pattern of excitatory output emerges; generally there are one or more glomeruli that change response type as well as a core of consistently responding glomeruli. **(D)** Mean percent of glomeruli responding as a function of stimulus loading from the blank (Bl) to neat (undiluted). Percentages are color coded blue, green, and red to indicate inhibitory, non-responsive and excitatory responses respectively. **(E)** Summary of responses of all glomeruli as a function of odor across stimulus loading. Each color coded box indicates which glomeruli (rows) responded consistently across all four loadings of a given odor (columns): Red, excited; blue, inhibited; green, non-responsive. CD indicates the response was concentration dependent. **(F)** Correlations between spatial patterns for different odors as a function of loading.

We next sought to determine if the spatial distribution of the virtual ensemble responses could account for changes in behavior at the defined psychophysical thresholds. Analyses were performed across a 500 ms response window starting at odor onset. Since each PN arborized a single addressable glomerulus, PN responses could be used as a spatial map of glomerular output and thus was used to quantify odor- and concentration-dependent spatial response patterns in the AL. Results displayed in Figure 6 are based on *z*-score transformed responses to each stimulus (relative to spontaneous spike rates).

Figures 6A,B are spatial representations of stimulus-driven responses for all concentrations of K6 and K8 respectively. By comparison, Figure 6C displays the blank response along with labels for represented glomeruli. As demonstrated above, behavioral data indicates that the lowest odor concentration (0.001 μg/2 μl) cannot be detected by *Manduca sexta* (see also Daly et al., 2007). Nevertheless there are differences in the spatial pattern elicited by the blank and the lowest concentrations of both odors. For example, glomerulus 34 is excited in response to the blank but inhibited by K6. In addition glomerulus 27 was inhibited by the blank but excited by both K6 and K8. The fact that subthreshold concentrations produce distinct spatial patterns confirms that spatial response patterns can vary in cases where the organism cannot perceive the presence or identity of the stimulus. At the behaviorally defined detection threshold (0.01 μg/2 μl), glomerulus 28 drops from the excitatory response for both odors, glomerulus 27 becomes inhibited in response to K8 whereas glomerulus 34 becomes active for K6. At the discrimination threshold (1.0 μg/2 μl) glomerulus 43 drops out of the excitatory representation for K6 and becomes inhibited, whereas in response to K8, glomerulus 28 again becomes excited. Interestingly, neat K6 elicits an excitatory response from most of the glomeruli, while neat K8 only modestly changes by eliciting an excitatory response from glomerulus 15 while losing the excitatory response to glomeruli 37 and 43. Table 2 indicates that across most odors a small number of glomeruli entered into or left the spatial response pattern and the amount of spatial change was reasonably consistent until neat odor is presented. Furthermore, the mean percentage of activated glomeruli did not increase substantially across the dilution series, varying between 50 and 53% from the blank to the discrimination threshold, whereas undiluted odor excited approximately 66% of glomeruli; this can be explained as a general recruitment of inhibited glomeruli which drops from 37 to 26% (Figure 6D). Overall these results are consistent with imaging data that indicate that as concentration goes up, there is an increase in the magnitude of the spatial response, but here we demonstrate that at psychophysical thresholds, this increase is nominal.

**Table 2:** **Difference in number of responding glomeruli to specific odorants presented at different concentrations**.

	Concentration comparisons					
	0.001	0.01	0.001	1.0	0.01	0.001
Odorant	0.01	1	1	neat	neat	neat
K6	3	2	3	7	5	4
A6	1	3	3	7	7	7
K8	2	1	1	5	6	4
A8	1	2	3	5	6	7
K10	0	0	0	5	5	5
A10	2	1	1	4	5	5
Mean	1.50	1.50	1.83	5.50	5.67	5.33

While some glomeruli produce variable output responses to an odor across concentration, others are consistent. This pattern of change vs. consistency is summarized by glomerulus and odor in Figure 6E. For example, across all concentrations of K6 (Figure 6E, column 1), a core group of glomeruli (16, 21, 27, 37, 44 and 53) were consistently excited, whereas 51 and 64 were consistently inhibited. The toroid (TO; a male-specific glomerulus) and glomerulus 63 (the labial pit glomerulus), were non-responsive to K6 at any concentration. The other seven glomeruli changed their responses in a concentration-dependent manner. By contrast, the core pattern for A6 (Figure 6E, column 2) was comprised of excited glomeruli 21, 28, 37 and 44, while glomeruli 2 and 64 were inhibited and 63 and the TO were non-responsive. Here, nine glomeruli showed concentration-dependent responses; that is they were responsive to some concentrations but not others. These results suggest that there may be a unique core group of glomeruli for each odorant, which manifest as a concentration-tolerant spatial “odor identity code”(Spors and Grinvald, 2002) and a secondary subset of glomeruli exhibiting concentration-dependent responses. As shown in Figure 6E however, A8 and A10 share a common core spatial pattern. Overall, whereas the percentage of activated glomeruli is fairly stable across the dilution series, this concentration dependent spatial heterogeneity outwardly appears to create an idiosyncratic spatial response pattern for each concentration across the dilution series; this heterogeneity may form the basis of a decorrelation between odors that could form a basis for discrimination thresholds. As shown in Figure 5D, concentration-dependency results in a systematic decorrelation between excitatory odor representations as a linear function of concentration (Figure 6F). Nevertheless, the spatial response patterns for odors do not become decorrelated until odorants are presented at neat concentration. This contrasts with the overall percentage of glomeruli representing odors, which does not change with increasing concentration until neat. Thus, while the percentage of responding glomeruli is stable the correlation drops in an approximately linear fashion. In either case, however, there is no notable change, given these analytic approaches, which indicates that as psychophysical thresholds are passed there is an observable shift in spatial response.

### Discrimination but not Detection Threshold Concentration Provides Added Spatiotemporal Separation of Odor Representations

The above analyses do not take into account the differences in response onset latencies as well as response durations. Thus, to determine whether these temporal features of responses contribute to a spatio-temporal representation that could account for behaviorally defined perceptual thresholds, a ED analysis was used. ED analysis uses the Pythagorean Theorem to geometrically assess the straight line distance between ensemble responses as a function of peri-stimulus time; because the ED analysis based on *z*-score normalized spike counts, the straight line distance between two high dimensional points can be described in statistical terms for each 20 ms step. Again, the spatial aspect is represented by 17 dimensions, where each dimension was defined by a single uniglomerular PN, and time was defined by each 20 ms step. We calculated the ED between responses to different stimuli for each concentration at each peri-stimulus time bin. Distance measures were normalized by the within repeat distance.

**Figure 7:**
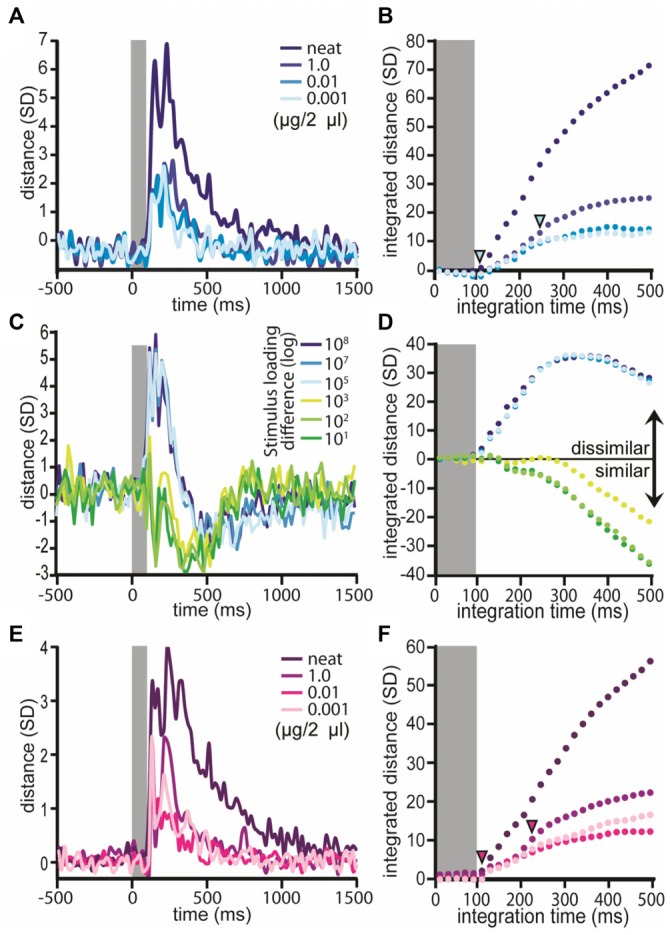
**Euclidean distance increases at discrimination threshold.** Normalized ED measures between the blank and different loadings of odor; results averaged across odors. Inset gray bar represents the stimulus. **(A)** Distance between the blank and odors at specific stimulus loadings; results displayed as a function of peristimulus response time. Each trace represents the mean distance of the population response to the blank and each loading. Note that each distance comparison peaks at or before 240 ms and all traces exceed 2 *SD*s. **(B)** Integrated distance over time for the comparisons shown in **(A)**. Inset arrowheads indicate when (in response time) there was a significant difference in integrated distance values between the lightest blue trace (i.e., ED between blank and the sub detection threshold concentration; loading = 0.001 μg/2 μl) and the darker blue trace (i.e., ED between the blank and discrimination threshold; loading = 0.001 μg/2 μl) or the darkest blue trace (ED between blank and neat odor). Significance was defined as a difference between the two comparison traces >2 *SD*s. **(C)** Normalized ED measures between loadings (within odor) as a function of response time (note blank responses are dropped from this analysis). Each trace represents the mean distance between all possible within-odor pairwise comparisons of different loading pairs. **(D)** Integrated distance between loadings. **(C,D)** Results displayed as a function of the absolute differences between loadings expressed as a log step differences. **(E)** Normalized ED measures between odors by stimulus loading as a function of response time (note blank responses are dropped from this analysis). Each trace represents the mean distance between all possible pairwise comparisons of odors for a given loading. **(F)** Integrated distance between odors by concentration. Inset inverted arrowheads indicate when a difference in integrated distance scores occurred. Here the comparisons were between the detection threshold concentration (loading = 0.01 μg/2 μl) and the discrimination threshold concentration (loading = 1.0 μg/2 μl) as well as neat odor.

First, we asked whether odor responses at each concentration provided additional information, relative to the blank-driven response, upon which the moth can recognize the presence of odor (i.e., odor detection). To achieve this, we calculated the ED between the blank response and responses from all six odors. Results of this analysis demonstrate that the responses to all concentrations diverge significantly from the blank (Figure 7A), producing peak distances between representations of 2.6, 2.6, 2.7 and 6.7 *SD*s for 0.001 μg/2 μl to neat respectively. This result suggests that there is potentially more information available at the level of the AL for “offline” statistical analysis than is accessible to the “live” moth at the level of sensory perception. Since the ED traces in Figure 7A are highly overlapped, this suggests that the detection threshold concentration does not provide added information that is detectable using ED analysis. However, because these distance measures vary over time, it is difficult here to assess whether there is any meaningful difference between the subthreshold and detection threshold concentrations that may accumulate over response time. Therefore, Figure 7B integrates distance as a function of time for each trace in Figure 7A and allows precise quantification of the accumulation of differences in distances generated by each comparison with the blank. By comparison, the sub-detection and detection thresholds do not separate significantly (i.e., >2 *SD*); this suggests that the neural ensemble responded in nearly the exact same way to odors presented at these two concentrations. By contrast, comparison of differences in integrated distance between the blank vs. sub-detection threshold and the discrimination threshold indicates that the discrimination threshold concentration provides added information in and above concentrations that cannot be detected; this is true of neat odor as well. Furthermore, with increasing concentration, the time required to meet the criterion for statistical difference is reached earlier (Figure 7B, inset arrowheads) but peaks later (i.e., ~150 vs. 180 ms post stimulus onset). Overall, these results imply that the spatiotemporal response elicited by odors at the detection threshold do not provide concentration-specific measureable information in the spatiotemporal representation.

To determine whether the same odor produced the same response at different concentrations, all possible within-odor between-concentration EDs were calculated (Figure 7C). Recall that across each step in concentration, shifts in spatial patterns were observed (Figure 6). Nevertheless, ED analysis revealed that through the dilution series, population responses were highly consistent as evidenced by a relative decrease in ED values (Figures 7C,D) by comparison to pre-stimulus spontaneous activity; this is consistent with previous reports (Stopfer et al., 2003). The exception to this were comparisons of the dilution series with undiluted odor. In these cases ED values increased starting at 120 ms indicating that the output representation for undiluted odors were substantially different. This difference between undiluted and the dilution series is consistent with increased percentage of responding outputs (see Figures 4, 6D), decreases in response latency (Figure 5G) and decreased spatial correlation (Figure 6F). We highlight however, that previous psychophysical characterization of odor discrimination as a function of concentration clearly demonstrates that moths differentially conditioned with discrimination threshold concentration are more able to differentially respond to those odors when presented undiluted odor. Thus the increase in spatial distribution of the response appears to not affect “odor object” constancy in moths *per se* (Mwilaria et al., 2008). Overall, Figure 7D establishes that as the absolute difference between concentrations decreases, there is a systematic decrease in integrated distance. For example, comparison of odor at one (sub detection vs. detection threshold) or two log step differences (detection vs. discrimination threshold) in concentration produced highly similar responses whereas comparison of concentrations that varied by three log steps (i.e., sub detection vs. discrimination threshold) were relatively less similar and comparison of any of the dilutions with undiluted odor (i.e., 4–7 log step differences) were substantially different.

Finally, to determine if there is added information present at the discrimination threshold, which is not present in the sub-discrimination threshold concentrations, we calculated the ED between all possible pairwise comparisons between odors for each concentration (note here that the blank responses are omitted from this analysis). Across all four concentrations of odor, EDs between representations of different odors within a concentration diverged rapidly, starting within ~90–100 ms and peaking between ~120–240 ms after stimulus onset (i.e., odor valve actuation; Figure 7E). Distance measures also produced two or more peaks; these can be traced back to differences in individual PN response onset latencies. Peak ED measures were 2.3, 2.3, 2.4 and 4.0 *SD*s for 0.001 μg/2 μl to neat respectively. Note that these peak ED values are smaller than those observed in comparison of the odors with blanks implying that the distance between the blank and the odors is greater than the distance between different odors. These peaks occurred within 120 to 240 ms of stimulus onset. The important question, however, is whether the discrimination threshold concentration produces a greater distance measure than the detection and sub-detection threshold concentrations. Thus we compared the difference in integrated EDs from the detection threshold concentration and the discrimination threshold and neat concentrations. This allowed us to determine if and at what point in response time there is added information produced by the discrimination threshold concentration (or above) that is in-and-above what is produced by the detection threshold concentration. As shown in Figure 7F the difference between integrated ED values for the detection and discrimination thresholds exceeds 2 *SD*s at 220 ms and comparison of detection threshold and neat even faster at 100 ms. These results indicate that there is added information at the concentration required for moths to identify odors that is not present at the detection threshold. This difference may provide a basis for perception of differences in odor identity and occurs within 220 ms of odor onset or about 120–140 ms after the initiation of the excitatory response.

## Discussion

The field of olfactory neuroscience has long focused on understanding the nature of odor representations but has not carefully considered how these representations relate to the actual ability of an organism to detect and discriminate odors. While there is considerable research assessing odor representations as a function of concentration, this is the first study to our knowledge that has attempted to directly tie physiological measures of olfactory representations to psychophysical detection and discrimination thresholds. By characterizing olfactory responses at these critical thresholds, we gain a deeper insight into the minimum spatial and temporal requirements necessary to produce salient and distinct odor representations. Keeping in mind that quantitative measures of differences between neural responses are assumed to relate to the animal’s internal neural representations, the goal of the present study was to identify shifts in the spatiotemporal components of odor responses at and around behaviorally defined thresholds for detection and discrimination. We statistically identified detection and discrimination thresholds using standard behavioral assays, then directly compared AL output representations of odors at stimulus concentrations below, at and above these critical thresholds. We then used analyses that focus on the spatial and the combined spatiotemporal aspects of responses; this allowed us to identify changes in representations that may account for behaviorally defined thresholds.

Spatial response patterns (as defined by mapping PN activity to specific glomeruli) were unique for each odorant and odor concentration. However, we were unable to identify any specific shifts in purely spatial activation patterns at either the detection or discrimination thresholds that might underlie shifts in perceptual salience. Furthermore, each odorant elicited a core of responding glomeruli that was concentration invariant. It is tempting to suggest that this core represents a basis for an “identity code”. However, these core patterns were present in response to concentrations below the discrimination threshold and in response to undiluted odor, all of which elicited responses from the core and typically one to several additional glomeruli; this suggests that core patterns are unlikely to represent an identity code *per se*. While the size of representations did not change within our dilution series (sans undiluted odor), the correlation between spatial patterns for different odors tended to systematically decrease as concentration increased, suggesting that it is not the core spatial pattern but rather the concentration dependent changes in the spatial patterns that provides information about odor identity. However, this decorrelation only became statistically significant with undiluted odors (about five orders of magnitude more concentrated than the discrimination threshold). Given that representation from only a third of all AL glomeruli was present in this study, it is possible that a greater percentage of AL coverage might yield a significant decorrelation at threshold values.

Spatiotemporal-based ED analysis (using a 20 ms temporal resolution) established that across a very brief response window (i.e., between 100–240 ms after odor onset) the output representation of an odor rapidly and significantly diverged from both the response generated by blanks and from among other odors; if we consider that the earliest evidence of a physiological response in the AL are subthreshold local field oscillations initiating ~60 ms after stimulus onset (Daly et al., 2011), this suggests it takes between 60–180 ms for the AL to both process and output an odor dependent code; thus the output code takes some time to optimize. The degree of divergence in the comparisons of odor with blank and between-odor comparisons was furthermore dependent on odor concentration as were spatial patterns. Representational separation of odor responses from the blank was approximately the same for both the sub detection and detection threshold concentrations. As with spatial patterns, this indicates that changes in the spatiotemporal output representations of the AL do not appear to provide added information as stimuli become perceptually salient (i.e., detectable) to the organism. Thus detection may rely on other mechanisms such as a shift in level of synchronization across the responding assembly (Schoppa and Westbrook, 2001; Lei et al., 2002), which is concentration-dependent (Christensen et al., 2000) but which virtual ensembles cannot assess and that could occur within the core glomeruli or more broadly.

Similarly, distances between the same odor at different concentrations for the dilution series did not yield positive distance and in fact when the differences between comparison concentrations was relatively small (i.e., 1–2 log steps) representations became relatively closer, relative to a three log step difference. Only in comparison with undiluted odor did distance become positive suggesting a unique concentration dependent response. We again highlight that moths appear to have no trouble discriminating undiluted odor. This suggests that the increase in the spatial size and decrease in spatial correlation, as well as the decrease in response latency of representations that is associated with very high stimulus concentrations, neither affects the moths ability to discriminate between odors (Mwilaria et al., 2008) nor do these effects associate with perceptual shifts in acuity. However, we note that honeybees do in fact perceive different concentrations as qualitatively different (Wright et al., 2005); currently, we have been unable to train moths to differentially respond to different concentrations of the same odor.

However, the difference in representational distance between odors at the discrimination threshold vs. at the detection threshold (below) was significant, indicating a relative increase (i.e., shift) in representational information, which could provide animals with a basis for making odor discriminations. Furthermore, this increased information at the discrimination threshold is manifest within ~100–240 ms post stimulus onset with higher concentration stimuli providing more information about odor identity faster. These results support the conclusion that odor discrimination is encoded in the spatiotemporal response.

There is a growing body of both behavioral and electrophysiological evidence that odor discrimination occurs rapidly (Uchida and Mainen, 2003; Daly et al., 2004b; Budick and Dickinson, 2006; Wesson et al., 2008a,b; Staudacher et al., 2009). Furthermore subtle manipulations of the temporal features of otherwise overlapped spatial maps can be exploited by the animal to make olfactory-based discriminations (Rebello et al., 2014) establishing that patterns of onset latencies of output responses from these networks are sufficient to explain odor discrimination. Finally, odor identification/discrimination occurs faster as task demands are decreased (Abraham et al., 2004; Rinberg et al., 2006) as would be the case at high stimulus intensities.

What drives the concentration-dependent heterogeneity of PN responses that is both observed in several species (Boeckh and Selsam, 1984; Kanzaki et al., 1989; Laurent et al., 1996; Hartlieb et al., 1997; Anton and Hansson, 1998; Müller et al., 2002; Reisenman et al., 2005), and which underlies stimulus specific spatiotemporal odor representations? The basis of these changes in PN responses across our dilution series must initiate at the level of the olfactory sensory array which establishes an initial spatial input pattern (Mombaerts et al., 1996; Wang et al., 1998; Vosshall et al., 2000). Furthermore OSNs can have differing transduction kinetics depending on the odor and OSN type (Spors et al., 2006; Su et al., 2011; Martelli et al., 2013) thereby adding temporal structure. Local interneurons (LNs) within the AL then presynaptically shape OSN response levels (Olsen and Wilson, 2008; Root et al., 2008; Olsen et al., 2010). This initial spatiotemporal input pattern is then further transformed as it passes to principle cells; this too is mediated by local lateral interactions. These lateral interactions which are mediated by LNs can be both inhibitory (Matsumoto and Hildebrand, 1981; Waldrop et al., 1987; Christensen et al., 1993; Wilson et al., 2004; Wilson and Laurent, 2005) and excitatory (Olsen et al., 2007; Root et al., 2007; Shang et al., 2007). LNs function to synchronize PN responses (Waldrop et al., 1987; Christensen et al., 1993, 1998b) and are the genesis of other forms of temporal structuring of output (Stopfer et al., 1997; MacLeod et al., 1998; Wehr and Laurent, 1999; Tripathy et al., 2010; Daly et al., 2011; Tabuchi et al., 2013). Thus there are several potential sources of the observed spatiotemporal response heterogeneity across odors and concentrations, all of which may play a role in establishing subsequent perceptual thresholds.

The notion of species specific glomerular odor input maps is well established in the insect AL and vertebrate OB. This map is based on a specific OSN projection pattern to the glomerular layer (Mombaerts et al., 1996; Wang et al., 1998; Vosshall et al., 2000). OSNs projecting to different glomeruli have different response properties but odor tuning can overlap across different OSN types (de Bruyne et al., 2001; Hallem et al., 2004; Hallem and Carlson, 2006). The activation of multiple glomerular input lines by the same odor is partially a consequence of overlapping odor tuning. Therefore, different odors and odor blends are represented by the activity of different sets of glomeruli, which overlap more for similar odors than for dissimilar odors (Friedrich and Korsching, 1997; Johnson et al., 1998, 2005; Galizia et al., 1999b; Sachse et al., 1999; Uchida et al., 2000; Fuss and Korsching, 2001; Wachowiak et al., 2002; Hansson et al., 2003; Kuebler et al., 2011). Consistent with this line of findings we observe highly overlapped, yet distinct groups of glomerular outputs, which responded to the structurally similar odors. In contrast to imaging studies, the odor response maps generated by virtual ensembles comprised a larger percentage of AL glomeruli (~13–30 vs. ~50% respectively; Staudacher et al., 2009). This discrepancy most likely relates to differences between methods (Galizia and Kimmerle, 2004), but possibly relates to differences between species. Currently, imaging methods are challenged to resolve ordinary (i.e., non pheromone) glomerular structures in *Manduca sexta* (Hansson et al., 2003; Bisch-Knaden et al., 2012; Kuebler et al., 2012), thus we are unable to make direct within species comparisons at this time. As mentioned, each odor excited a unique core group of glomeruli across concentration, while other glomeruli were concentration dependent. However, the percentage of activated glomeruli increased little (~3%) across the dilution series, only increasing significantly for undiluted odors. Imaging studies across several species also indicate that glomerular activation patterns expand with increasing concentration (Fuss and Korsching, 2001; Wachowiak et al., 2002; Sachse and Galizia, 2003; Wachowiak and Cohen, 2003; Strauch et al., 2012) but this expansion likely occurs well above the concentrations required by the animals to discriminate. This difference in results across methods is ultimately important but difficult to assess without corresponding psychophysical data. Imaging studies also suggest that spatial response information alone is sufficient to discriminate odors and odor concentrations (Linster et al., 2001; Sachse and Galizia, 2003; Galán et al., 2004), however with one exception (Linster et al., 2001), these studies have not been tightly coupled to behavioral evidence of discrimination and in this later case, discrimination thresholds were not quantified. In conclusion then, our results demonstrate that spatio-temproal representations change as a function of concentration, even at concentrations not detectable by the animal. Furthermore, odor discrimination occurs, not as a result of expanding spatial maps but rather changing spatiotemporal dynamics of the primary olfactory network that evolve within ~120–140 ms from the initiation of the excitatory response; thus space takes time. Finally and perhaps most importantly, our results highlight the importance of tying behaviorally defined thresholds to neurophysiological measures to accurately quantify what is necessary and sufficient for perceptually salient odor representations to be formed.

## Author Contributions

KCD conception and design of research; EMS performed intracellular experiments; KCD and PDC performed the psychophysical experiments; KCD, SB and EMS analyzed data; RT identified glomeruli; KCD, SB and EMS prepared figures; KCD drafted, edited and revised manuscript; RT and JS, SB and PDC commented on the manuscript; all authors approved final version of manuscript.

## Conflict of Interest Statement

The authors declare that the research was conducted in the absence of any commercial or financial relationships that could be construed as a potential conflict of interest.
